# Development of Fluorine-Free Electrolytes for Aqueous-Processed Olivine-Type Phosphate Cathodes

**DOI:** 10.3390/molecules29194698

**Published:** 2024-10-04

**Authors:** Claudia Limachi, Klaudia Rogala, Marek Broszkiewicz, Marta Cabello, Leszek Niedzicki, Michel Armand, Władysław Wieczorek

**Affiliations:** 1 Faculty of Chemistry, Warsaw University of Technology, 00-664 Warsaw, Poland; 2 Centre for Cooperative Research on Alternative Energies (CIC energiGUNE), Basque Research and Technology Alliance (BRTA), Alava Technology Park, Albert Einstein 48, 01510 Vitoria-Gasteiz, Spain; 3 Alistore-European Research Institute, CNRS FR 3104, Hub de l’Énergie, 80039 Amiens, France

**Keywords:** fluorine-free lithium salts, lithium-ion batteries, green chemistry, sustainability, aqueous electrodes, aqueous binders

## Abstract

Environmental impacts and resource availability are significant concerns for the future of lithium-ion batteries. This study focuses on developing novel fluorine-free electrolytes compatible with aqueous-processed cobalt-free cathode materials. The new electrolyte contains lithium 1,1,2,3,3-pentacyanopropenide (LiPCP) salt. After screening various organic carbonates, a mixture of 30:70 wt.% ethylene carbonate and dimethyl carbonate was chosen as the solvent. The optimal salt concentration, yielding the highest conductivity of 9.6 mS·cm^−1^ at 20 °C, was 0.8 mol·kg^−1^. Vinylene carbonate was selected as a SEI-stabilizing additive, and the electrolyte demonstrated stability up to 4.4 V vs. Li+/Li. LiFePO_4_ and LiMn_0.6_Fe_0.4_PO_4_ were identified as suitable cobalt-free cathode materials. They were processed using sodium carboxymethyl cellulose as a binder and water as the solvent. Performance testing of various cathode compositions was conducted using cyclic voltammetry and galvanostatic cycling with the LiPCP-based electrolyte and a standard LiPF6-based one. The optimized cathode compositions, with an 87:10:3 ratio of active material to conductive additive to binder, showed good compatibility and performance with the new electrolyte. Aqueous-processed LiFePO_4_ and LiMn_0.6_Fe_0.4_PO_4_ achieved capacities of 160 mAh·g^−1^ and 70 mAh·g^−1^ at C/10 after 40 cycles, respectively. These findings represent the first stage of investigating LiPCP for the development of greener and more sustainable lithium-ion batteries.

## 1. Introduction

Transitioning towards a carbon-neutral society presents various challenges, including those in electrochemical energy storage. Since the 1990s, lithium-ion batteries have been the most common commercial energy storage device. However, current commercial lithium-ion batteries (LIBs) use fluorinated salts, solvents, and electrolyte additives. The main hazards of fluorinated compounds include potential toxicity, a negative environmental impact, and complicated recycling [1,2,3].

The most commercial electrolyte uses the fluorinated salt lithium hexafluorophosphate (LiPF_6_), which contains 75 wt.% fluorine, where the PF_6_^−^ anion can release toxic substances like HF and POF_3_ in the presence of moisture, high temperatures, or acids [4,5,6]. Although fluorine-containing electrolytes are essential for optimal performance [1,7], alternatives without fluorine have been explored. Salts such as lithium perchlorate (LiClO_4_), which offers high solubility in aprotic solvents, high conductivity, and good electrochemical stability, have been explored. However, the high oxidation state of Cl^VII^ makes it highly explosive in organic solutions [8,9]. Lithium tetrachloroaluminate (LiAlCl_4_) is another fluorine-free lithium salt that has been extensively studied, but side reactions forming LiCl can block electrode reactions [10]. Additionally, AlCl_4_^−^ oxidizes at 4 V, low for many applications. For the half-cell configuration for Li/LFP at 1C in the potential range of 0–2 V, the specific capacity obtained for LiAlCl_4_ 3SO_2_ is 148 mAh·g^−1^.

Lithium bis(oxalate)borate (LiBOB) has high electrochemical (>4.5 V vs. Li^+^/Li) and thermal stability, but its solubility in the EC:EMC mixed solvent system is below 0.9 mol kg^−1^ solvent and its conductivity is low, being 2–3 mS cm^−1^ at 25 °C [11,12,13,14,15]. The specific capacity of LiBOB was found to be in the range of 90–120 mAh·g^−1^ in GBL and 135 mAh·g^−1^ in PC:EMC:DMC at room temperature. Adding to that, in the half-cell Li/LFP cell configuration at C/10 in the range potential of 2.5–4.4 V and at room temperature, a specific capacity of 165 mAh·g^−1^ in LiDCTA:PC, 158 mAh·g^−1^ in LiDCTA:PEGDME, and 165 mAh·g^−1^ in LiB(CN)_4_:PEGDME was found [6]. Therefore, despite some previously reported fluorine-free lithium salts, most do not meet the commercial electrolyte requirements [16,17]. On the anion side, weakly coordinating FSI (FSO_2_NSO_2_F), TFSI (CF_3_SO_2_NSO_2_CF_3_), and “Hückel-type anions” like 2-trifluoromethyl-4,5-dicyanoimidazole (TDI) have been developed for Li-ion cells, yet all still contain fluorine [18,19].

**Figure 1:**
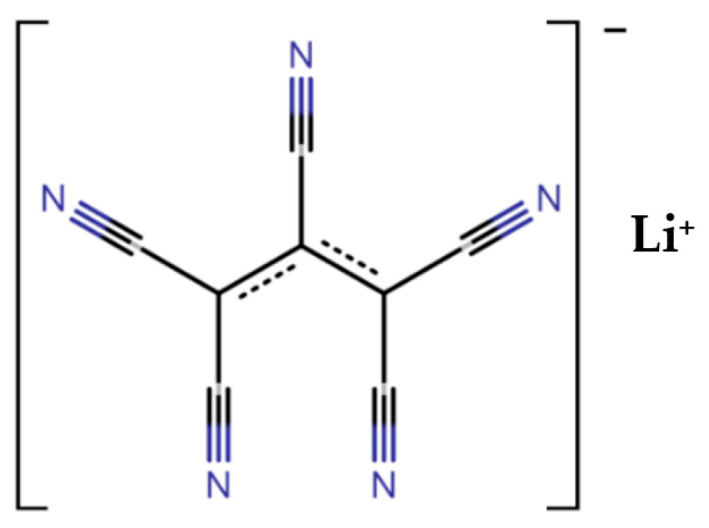
Chemical structure, lithium 1,1,2,3,3-pentacyanopropenide (LiPCP), novel fluorine-free lithium salt for electrolytes.

This work presents a novel fluorine-free alternative to existing salts for Li-ion battery electrolytes, lithium 1,1,2,3,3-pentacyanopropenide (LiPCP, Figure 1). We found that 1,1,2,3,3-pentacyanopropenide (PCP) is among the most stable carbanions. Its remarkable stability is attributed to the inductive effect of the five electron-withdrawing cyano groups. Consequently, the negative charge is delocalized across the central propenide structure [20,21]. R. H. Boy reported that no changes in the spectra of PCP were noted up to 11 M perchloric acid or ~85% sulfuric acid, indicating that the free anion may still exist even in this highly acidic environment [22], making this salt stable and with no side reactions in contact with air, water, and even highly acidic solutions. Furthermore, this novel lithium salt presents high thermal stability up to >300 °C [23].

The new generation of lithium-ion batteries targets sustainable and greener chemistries, with a special focus on cobalt-free cathode materials. Critical raw materials such as cobalt (Co) and nickel (Ni) are classified as carcinogenic, mutagenic, and toxic to reproduction (CMR) [24]. Cobalt, in particular, is a major cost driver and raises significant moral and environmental concerns due to questionable mining conditions [25,26,27,28]. There are, however, alternative electrode materials that do not include critical resources. Phospho-olivine-type cathode materials like LiFePO_4_ (LFP) and LiMn_x_Fe_1−x_PO_4_ (LMFP) with 0 ≤ x ≤ 1 are considered eco-friendly, since they consist solely of abundant elements [29,30]. Furthermore, the high structural stability of the polyanionic phosphate network and the relatively low operational voltage window, which prevents unwanted parasitic reactions of the battery electrolyte, allow a long cycle and span life for olivine-based LIBs [31,32,33].

Fluorinated compounds are not only in electrolytes, but also in electrodes, with fluorinated binders like polyvinylidene fluoride (PVdF), which involves the use of N-methyl-2-pyrrolidone (NMP) as a solvent, both used in most commercial electrodes. The binder plays an important role in the performance, cost, environmental impact, and recycling possibility of the battery [34,35]. At present, standard electrode processing uses PVdF as a binder. Despite its good electrochemical stability and adhesion ability [36,37,38,39], it is expensive and makes the battery recycling process problematic. In fact, during electrode manufacturing, this binder is dissolved into the hazardous, teratogenic, and irritating solvent N-methyl-2-pyrrolidone (NMP), [40,41,42]. For this reason, water-soluble binders are gaining more attention. Among these, sodium carboxymethyl cellulose (CMC-Na) has been considered as a potential binder for lithium-ion batteries. CMC provides several advantages, including being economically available, hydrophilic, environmentally friendly, biodegradable, and compatible with potential electrode materials to develop green and sustainable LIBs [43,44,45,46].

A SEI-stabilizing electrolyte additives, vinylene carbonate (VC) and acetonitrile (AN), both fluorine-free, are proposed as alternatives to other popular additives like fluoroethylene carbonate (FEC), which is the most widely used commercial electrolyte additive [47,48]. VC is reductively decomposed or oxidized prior to other electrolyte components [49,50]. AN has a low cost, and its low viscosity and very high dielectric constant make it a good option for dissolving salts [51,52].

Additionally, the growing awareness and concern about the environmental and health impacts of per- and polyfluoroalkyl substances (PFAS) have led to significant regulatory actions [53]. For instance, the European Union has proposed a comprehensive ban on all non-essential PFAS by 2025 and across all uses by 2030 [54]. Similarly, the United States Environmental Protection Agency (EPA) has been working on regulations to limit PFAS in industrial uses. Canada is also following suit, with initiatives to restrict these substances under the Canadian Environmental Protection Act. These bans highlight the urgent need to develop sustainable alternatives in various industries, including energy storage. In this context, the development of fluorine-free batteries and components becomes not only a technological advancement, but also a crucial step towards a safer and more sustainable society.

Therefore, in this work, we report the viability of this new fluorine-free lithium salt, LiPCP with fluorine-free electrolyte additives working with aqueous-processed LFP and LMFP cathodes. For this purpose, we report the electrolyte conductivity at different concentrations and temperatures, an analysis of the electrolyte stability, and an analysis covering voltammetry and galvanostatic cycling, demonstrating the capability of fluorine-free electrolytes for aqueous-processed olivine-type cathode materials. These findings represent the initial phase of investigating safer and more environmentally friendly electrolytes with aqueous-processed electrodes for lithium-ion batteries.

## 2. Results

### 2.1. Electrochemical Properties—Ionic Conductivity and LSV

Finding the most suitable organic carbonate solvent mixture (EC, DMC, DEC, EMC) for the LiPCP salt was the first step of electrolyte optimization. To do so, ionic conductivity measurements were carried out. For comparison purposes, mixtures of solvents containing LiPF_6_ were prepared.

**Figure 2:**
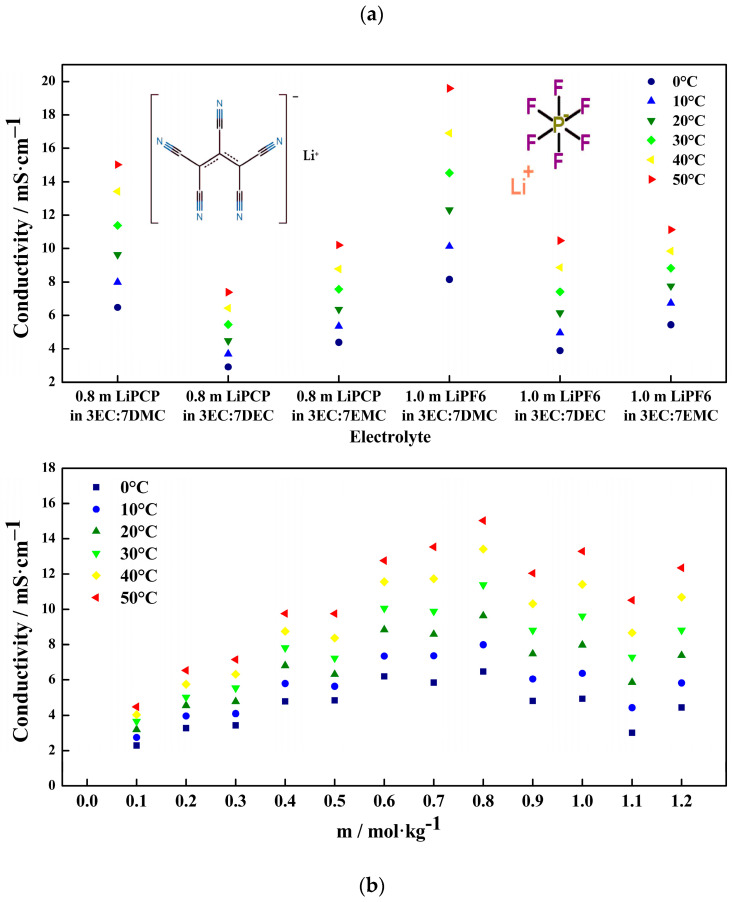
(**a**) Ionic conductivity of LiPCP and LiPF_6_ in various organic carbonate solvent mixtures from 0 to 50 °C. (**b**) Ionic conductivity of LiPCP in EC:DMC (30:70 wt.%) at concentrations in the range of 0.1–1.2 mol·kg^−1^ from 0 to 50 °C.

Figure 2a shows the ionic conductivity of LiPCP and LiPF_6_ in different organic carbonate solvents and at different temperatures. Solutions of 0.8 mol·kg^−1^ of LiPCP in EC:DMC (30:70 wt.%), EC:DEC (30:70 wt.%), and EC:EMC (30:70 wt.%); and 1.0 mol·kg^−1^ of LiPF_6_ in EC:DMC (30:70 wt.%), EC:DEC (30:70 wt.%), and EC:EMC (30:70 wt.%) were investigated. The highest conductivities of LiPCP and LiPF_6_ were achieved with the mixture of EC:DMC. At a temperature of 20 °C, LiPCP in EC:DMC has a conductivity of 9.6 mS·cm^−1^, and LiPF_6_ in EC:DMC has a conductivity of 12.3 mS·cm^−1^, and the difference between them was of ca. 28% in favor of LiPF_6_ solution.

The behaviors of LiPCP and LiPF_6_ with different mixtures of solvents have the same tendency, that is, having the highest conductivities with EC:DMC and the lowest conductivities with EC:DEC. This can be attributed to the lower viscosity of DMC and the highest of DEC, out of the three tested linear carbonate solvents. Therefore, the mixture of LiPCP in EC:DMC was considered as the baseline electrolyte for further experiments in this work. The conductivity values of each electrolyte, as well as the difference in percentage between the LiPF_6_- and LiPCP-salt-containing electrolytes, can be found in Table S1 and Table S2, respectively.

Figure 2b and Table S3 show the ionic conductivity of LiPCP at different temperatures and molal concentrations in the range of 0.1–1.2 mol·kg^−1^ in EC:DMC (30:70 wt.%). At 20 °C, it can be seen that the highest LiPCP conductivity was reached at 0.8 mol·kg^−1^, with a value of 9.6 mS·cm^−1^. From Table S3, it can be inferred that the conductivity of LiPCP solutions satisfies the minimal conductivity requirements of lithium-ion batteries, with typical conductivities in the 5–10 mS·cm^−1^ range [55]. It should also be noted that the optimal concentration of LiPCP is lower (0.8 mol·kg^−1^) than the optimal concentration of LiPF_6_ (1.0 mol·kg^−1^), which is beneficial from an economic and ecological point of view, as less salt is required. Therefore, displaying high mobility at a lower concentration, and resulting in an acceptable ionic conductivity, LiPCP shows its competitiveness compared to the conventional LiPF_6_-based electrolytes.

**Figure 3:**
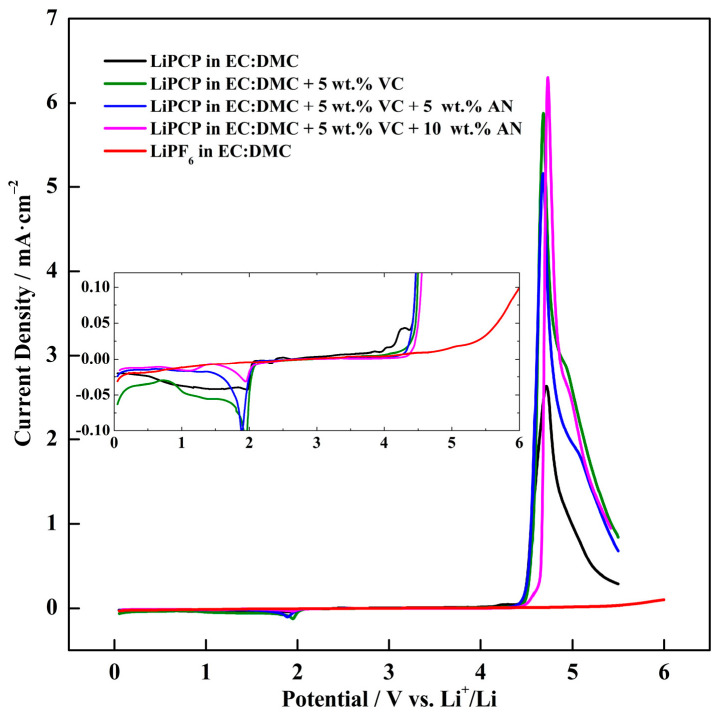
Linear sweep voltammetry (LSV) of 0.8 mol·kg^−1^ LiPCP-based electrolytes without additives and with various electrolyte additive concentrations, with the Pt disc as the working electrode and the Li metal disc as the reference electrode in a Swagelok-cell system, at a scan rate of 0.5 mV·s^−1^. For comparison purposes, data on a LiPF_6_ electrolyte are included. The inset shows the zoomed area.

In addition, the electrochemical stability of the baseline LiPCP electrolyte (0.8 mol·kg^−1^ LiPCP in EC:DMC (30:70 wt.%)) was investigated by LSV. Quantum chemistry calculations can classify the anodic stabilities based on the anion structure and functional groups. However, the concentration of Li^+^ and solvents influence the anion stability [6,56]. That is why it is important to study the impact of different additives like VC and AN on the electrolyte’s electrochemical stability. Figure 3 shows the voltammograms of the electrolyte without additives, with 5 wt.% of VC, and with 5 wt.% of VC plus either 5 wt.% or 10 wt.% of AN. It can be noticed that the lowest stability was exhibited by the electrolyte without additives, which was limited to 4.0 V vs. Li^+^/Li. By adding 5 wt.% of VC to the electrolyte, the stability window increased up to 4.4 V vs. Li^+^/Li. The addition of 10 wt.% AN further increased this window (4.45 V vs. Li^+^/Li). There is also a reduction observed in the nitrile-based electrolyte at around 2 V, however, the origin has not been determined. The anion could be decomposed on the electrode surface or be part of the solvents/electrolyte additive decomposition, resulting in reactions/products difficult to predict. Therefore, the experimentally determined electrolyte stability, measured in a Li/Pt system, should be considered as a reference of the electrolyte stability in cells with active electrode materials. The results suggest that the stability window of the screened electrolytes is suitable for working with cobalt-free commercial electrodes such as LFP. Such an electrochemical stability window also provides the possibility to work with LMFP.

### 2.2. Lithium Passivation

**Figure 4:**
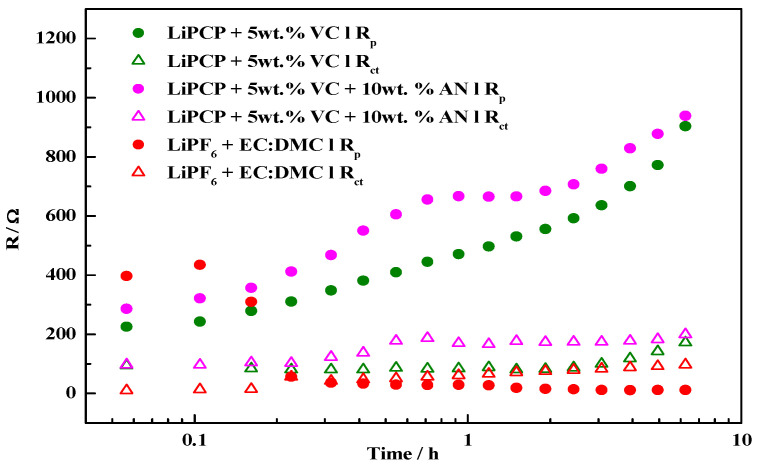
Passive layer resistances (R_p_) and charge transfer resistances (R_ct_) for Li|electrolyte|Li cells with three different electrolytes.

The results observed for the samples reflect processes occurring both at the anode and the cathode. It creates particular problems when trying to compare data obtained for samples with different electrolytes. In such cases, the variation in the resistance of the passivation layer on the lithium electrode may take prevalence over the effect on the cathode side. In order to gauge its impact, lithium passivation tests were carried out. The charge transfer resistance (R_ct_) and passive layer resistance (R_p_) obtained from the fitting of impedance spectra (Figure S1) are presented in Figure 4. The R_p_ of lithium electrodes in LiPCP-based electrolytes increases in the span of 6 h from ca. 200 Ω to ca. 1000 Ω. For the sample with LiPF_6_, exhibiting similar resistance at the beginning, R_p_ drops within the first hour to below 100 Ω, indicating the dissolution of a native passivation layer on lithium and the formation of a new one of much lower resistance. The R_ct_ values are in a similar range for all of the samples, increasing to ca. 100 Ω for the LiPF_6_ sample and ca. 200 Ω for LiPCP sample. The resistance of the LiPCP + 5 wt.% VC + 10 wt.% AN sample exhibits slightly higher values in comparison to the analogous sample without AN.

This can be explained by the reactivity of AN towards metallic lithium [57]. However, it was also shown that the presence of VC allows for the creation of stable SEI for AN-containing electrolytes [58]. Higher resistance for LiPCP-containing samples will likely translate to higher polarization of the cells during cycling, with the said effect being independent of the performance of electrolyte or cathode material.

### 2.3. Cyclic Voltammetry

As one of the objectives for this research is to work with sustainable electrode materials, LFP and LMFP were selected as the cathode materials used to test the electrolyte/electrode reversibility. For this purpose, CV measurements were carried out. Based on the previous LSV results, the electrolyte of choice was 0.8 mol·kg^−1^ LiPCP in EC:DMC (30:70 wt.%) with 5 wt.% of VC, due to its sufficient stability window for working with both LFP and LMFP electrodes. Regarding the LFP material, different water-based electrode formulations were screened by means of CV (Table 1).

**Figure 5:**
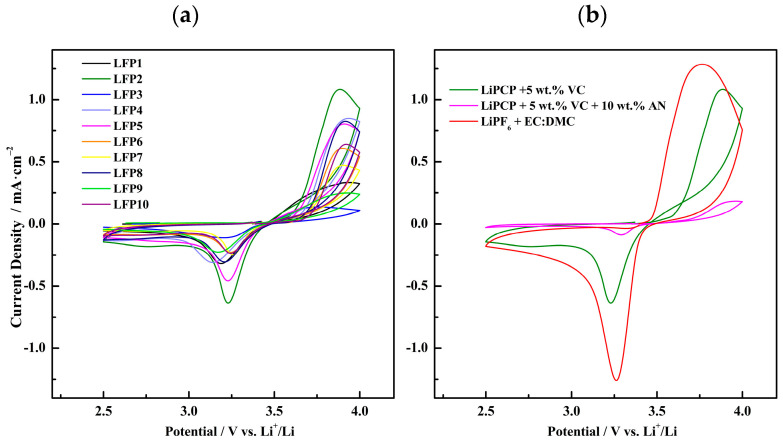
(**a**) CV voltammograms (1st cycle) of corresponding Li/LFP cells at different LFP compositions, with 0.8 mol·kg^−1^ LiPCP in EC:DMC (30:70 wt.%) and 5 wt.% of VC at a scan rate of 0.5 mV·s^−1^ at room temperature. (**b**) CV voltammograms (1st cycle) of corresponding Li/LFP2 cells with 0.8 mol·kg^−1^ LiPCP in EC:DMC (30:70 wt.%) with 5 wt.% VC and with 5 wt.% VC plus 10 wt.% AN. For comparison purposes, data with the reference LiPF_6_ electrolyte are shown. Tests were carried out with a scan rate of 0.5 mV·s^−1^ at room temperature.

Table 1 also lists the difference between the anodic and cathodic peaks potential (ΔV) for each electrode composition. It is correlated to the polarization or reversibility of the redox reaction, the smaller the difference, the greater the reversibility, and the smaller the polarization. Figure 5a shows the reversible redox reactivity of the aqueous-processed LFP cathode at different compositions prepared with the CMC binder and H_2_O as a solvent, with the LiPCP-based electrolyteThe potential difference between oxidation and reduction peaks is an indicator of the electrochemical kinetics. The LFP2, LFP3, LFP6, and LFP7 samples show the smallest potential difference and peak area ratio. Interestingly, LFP2 shows the sharpest peaks and the highest current density, which indicates the fast kinetics of the redox process. Furthermore, the potential difference (ΔV) is only 0.65 V, denoting relatively low polarization. These results indicate that the composition of LFP2 working with the LiPCP-based electrolyte allows us to obtain better conductivity compared to the other compositions. This behavior can be attributed to the CM/binder ratio. When the ratio is higher than 3.3, a higher content of conductive material is present in the slurry, which does not allow a uniform distribution of the active material, resulting in agglomerations. Additionally, the low presence of the binder in the slurry produces a low cohesion between the active and conductive materials; moreover, a low adhesion between the slurry and the current collector produces a poor performance. Adding to that, when the ratio is lower than 3.3, less conductive material is present in the electrode formulations, causing a lower reversibility and a higher polarization during the cycling process.

Subsequently, having in mind the good result for the electrolyte with 10 wt.% AN (Figure 3), CV was performed with that electrolyte and LFP2 cathode (Figure 5b). No improvement, with respect to the electrolyte without AN, was found here. In fact, lower current densities are achieved. The sample with AN showed ca. 10 times lower current density and the highest polarization, therefore, the worst performance. The highest polarization and the lowest current density are consistent with the highest resistance obtained in passivation tests. Also, the magnitude of the current drop for this sample might indicate some other problem with compatibility between the AN-containing electrolyte and the used cathode. A potential explanation is that AN can be easily deprotonated, followed by polymerization in contact with lithium metal and, consequently, the stability of the AN-containing half-cell is quite poor. Working with the same electrode formulation and LiPF_6_, a potential difference of 0.38 V was observed. Although the peak area ratio is not perfect, hinting at significant irreversible reactions of LiPF_6_, the sample showed by far the highest current density. This could be due to the lower resistance of the passivation layer on lithium.

Table 2 details the LMFP cathode fabrication at different water-based electrode formulations by means of CV. Super P carbon black and Ketjenblack EC600JD carbon black were used as the conductive materials. CMC solutions with 1 wt.% or 1.5 wt.% of CMC were used as the aqueous binder for the slurry preparation.

**Figure 6:**
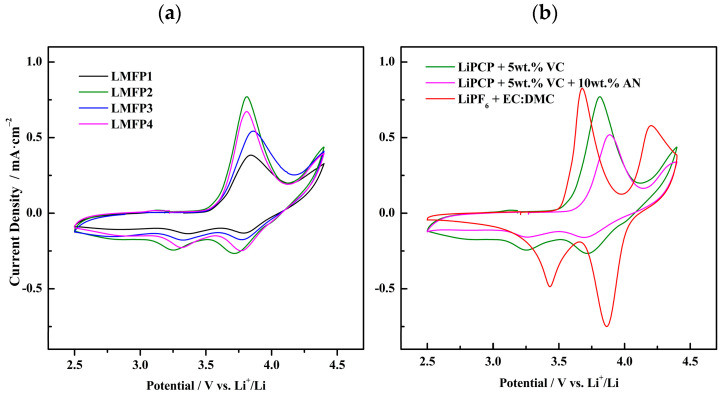
(**a**) CV voltammograms (1st cycle) of corresponding Li/LMFP cells at different LMFP compositions, with 0.8 mol·kg^−1^ LiPCP in EC:DMC (30:70 wt.%) and 5 wt.% of VC at a scan rate of 0.5 mV·s^−1^ at room temperature. (**b**) CV voltammograms (1st cycle) of Li/LMFP2 cells with 0.8 mol·kg^−1^ LiPCP in EC:DMC (30:70 wt.%) with 5 wt.% of VC and with 5 wt.% VC plus 10 wt.% AN. For comparison purposes, data with a reference LiPF_6_ electrolyte are shown. Tests were carried out with a scan rate of 0.5 mV·s^−1^ at room temperature.

Figure 6a displays the CV curves of the fabricated LMFP cathodes with the LiPCP electrolyte that contains 5 wt.% of VC. We can see the oxidation and reduction peaks of Fe^3+^/Fe^2+^, whereas Mn^3+^/Mn^2+^ is not fully visible, due to the potential cut-off. In terms of the potential difference between the anodic and cathodic peaks of Mn and Fe, they were slightly shifted from each other. Table 2 presents the potential difference in Fe for the LMFP1, LMFP2, LMFP3, and LMFP4 samples, and they were equal to 0.47 V, 0.55 V, 0.53 V, and 0.49 V, respectively. The peak current density for LMFP2 was 0.77 mA·cm^−2^, which is visibly higher than that of the rest, which indicates fast kinetics of the redox processes. In addition, the narrower peaks of LMFP2 in comparison with the other LMFP samples suggest a lower polarization and much better reversibility. Therefore, LMFP2 exhibits the fastest Li^+^ diffusion and the highest reversibility. It is worth mentioning that fast electrode reaction kinetics and high reversibility should result in superior cyclability. This can be attributed to the porous characteristic of Ketjenblack, which improves the transport of electrons in the electrode, resulting in a better reversible process [59]. LMFP1 and LMFP2 have the same compositions, varying in the conductive material only, where LMFP2 shows better performance; moreover, the same happens when we compare LMFP3 and LMFP4, where LMFP4, when working with Ketjenblack, shows a higher and narrower peak than LMFP3. Therefore, the conductive material effect on the electrode performance is quite noticeable.

Figure 6b shows the optimized aqueous-processed LMFP2 electrode working with LiPCP in EC:DMC (30:70 wt.%) with 5 wt.% VC and 5 wt.% VC with 10 wt.% AN content, as well as the LiPF_6_-based electrolyte. In terms of potential differences, 0.57 V and 0.64 V values were obtained, corresponding to the oxidation/reduction of Fe for 5 wt.% VC and 5 wt.% VC with 10 wt.% AN content, respectively. Exhibiting similar behavior to that of an analogue LFP system, LMFP with 5 wt.% VC and 10 wt.% AN content has shown inadequate cell cyclability and stability. This is attributed to the AN reaction with lithium metal during cycling. It results in the highest potential difference, showing stronger polarization and worse reversibility. The sample with LiPF_6_ showed the lowest polarization and the highest current density, which might be of particular importance for this cathode, due to the low margin of potential over the Mn^3+^/Mn^2+^ plateau.

### 2.4. Rate Capability

The performance of the fluorine-free LiPCP electrolyte with the aqueous-processed LFP and LMFP electrodes was further tested by galvanostatic means in half-cell coin cells.

**Figure 7:**
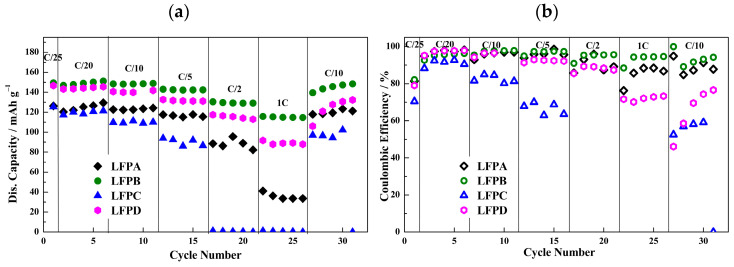
Rate performance of Li/LiFePO_4_ cells with 0.8 mol·kg^−1^ LiPCP in EC:DMC (30:70 wt.%) and 5 wt.% of VC. Potential range: 2.5–3.9 V vs. Li^+^/Li. (**a**) Discharge capacities in mAh·g^−1^. (**b**) Coulombic efficiency in (%).

Motivated by the results obtained for the LMFP material in the screening of the carbon additive and binder solution concentration, the same screening was performed for the LFP material. In Table 3, it is important to note that the electrode formulation ratios (AM:CM:binder to 87:10:3) were maintained as in the LFP2 sample from the cyclic voltammetry section. The composition labeled as LFPA corresponds to this initial formulation, which was used for cyclic voltammetry studies. The primary variations in the subsequent samples involved changes in the carbon black (conductive material) and CMC concentration in the solution, with the effects of these changes being presented in Figure 7. Due to the positive results from using ketjenblack for LMFP, this carbon black was also incorporated into the optimization of the LFP electrodes. Additionally, the impact of different CMC concentrations on cell performance was tested, with 1 wt.% and 1.5 wt.% concentrations selected for further optimization of LFP electrode fabrication.

Figure 7a exhibits the performance at different C rates of the cells using the various aqueous-processed LFP electrode formulations. LFPA and LFPC displayed low specific capacities, lower stability, and lower coulombic efficiencies compared to the rest of the LFP samples. The replacement of Super P by Ketjenblack in the electrode formulation had a strong impact on cell performance. It led not only to a higher initial capacity (125 mAh·g^−1^ for LFPA and LFPC vs. 150 mAh·g^−1^ for LFPB and LFPD), but also to higher overall capacities at different C rates. This difference is especially evident at fast rates like 1C. Moreover, fewer fluctuations in the capacity values were observed for cathodes with Ketjenblack (LFPB and LFPD), and higher coulombic efficiency values were achieved, reaching above 90% for all of the C rates in the case of LFPB (Figure 7b). Ketjenblack seems to provide better electrical contact for AM particles, which is of particular importance for the AM of relatively low conductivity [60]. The initial coulombic efficiency values for LFPA, LFPB, LFPC, and LFPD were 81.4%, 82.1%, 70.4%, and 79.0%, respectively. These relatively low values can be attributed to the instability of the lithium anode and continuous passivation of the evolving lithium surface. Nevertheless, they are good values considering that the standard LiPF_6_-based electrolyte has been replaced by a new chemistry. Regarding the concentration of CMC in the binder solution, LFPB (1 wt.%) surpasses the performance of LFPD (1.5 wt.%), showing much higher coulombic efficiency, regardless of the CM used. Therefore, it can be tentatively suggested that the higher stability and capacities shown by LFPB can be attributed to a better homogenization of the slurry, which is, in turn, due to a lower viscosity of the CMC binder solution. Therefore, a homogeneous distribution occurs between the active and conductive materials, resulting in a better cohesion among the particles. Additionally, a better adhesion between the slurry and the current collector is given by the composition, where the properties have a direct effect on the cell performance.

**Figure 8:**
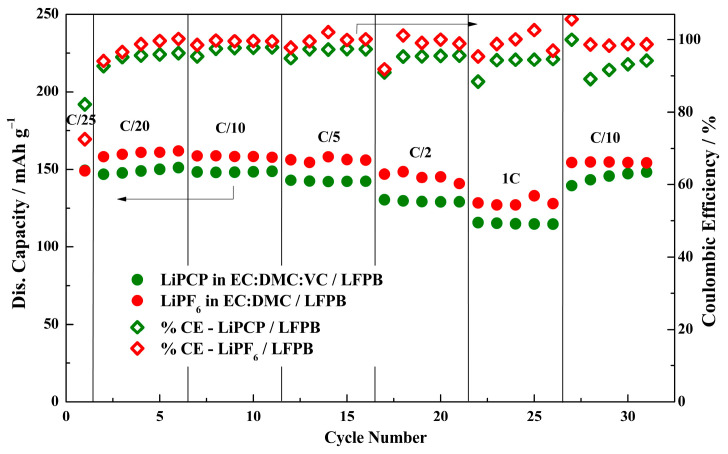
Rate performance and coulombic efficiency of Li/LiFePO_4_ (LFPB) cells with 0.8 mol·kg^−1^ LiPCP in EC:DMC (30:70 wt.%) and 5 wt.% of VC; and with 1.0 mol·kg^−1^ LiPF_6_ in EC:DMC. Potential range: 2.5–3.9 V vs. Li^+^/Li.

Furthermore, the comparison of the best-performing electrode (LFPB) with the standard LiPF_6_-based electrolyte (Figure 8) led to interesting results. When comparing the performance of LFPB in LiPCP and LiPF_6_ electrolytes, it can be noticed that the same initial discharge capacity (150 mAh·g^−1^) was achieved. Surprisingly, the initial coulombic efficiency obtained was slightly lower (72.5% vs. 82.1%) when using LiFP_6_. The coulombic efficiency was stable and above 90% for both electrolytes in the subsequent cycles. While it is true that the overall capacity and coulombic efficiency values were lower when using the LiPCP electrolyte, these values did not fluctuate as much as they did for the case of the LiPF_6_ electrolyte. When analyzing the charge–discharge curves (Figures S2 and S3), it can be seen that both samples exhibit stable behavior, with a slightly higher polarization for the sample with LiPCP. It does not significantly impact the capacity and rate performance of the cell. Overall, LFP electrodes are compatible with the LiPCP-based electrolytes, showing adequate performance.

**Figure 9:**
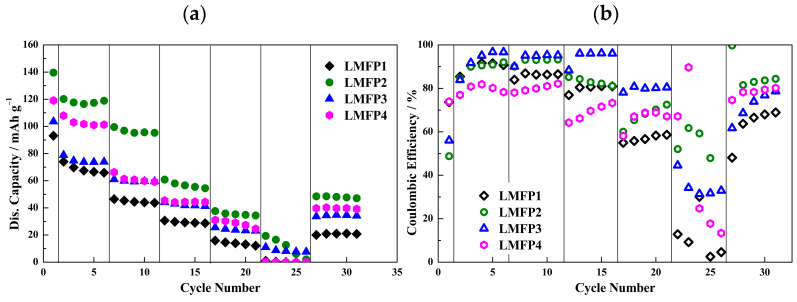
Rate performance of Li/LiMn_0.6_Fe_0.4_PO_4_ cells with 0.8 mol·kg^−1^ LiPCP in EC:DMC (30:70 wt.%) and 5 wt.% of VC. Potential range: 2.4–4.2 V vs. Li^+^/Li. (**a**) Discharge capacities in mAh·g^−1^. (**b**) Coulombic efficiency in percentage (%).

Figure 9a shows the performance at different C rates of the cells using the various aqueous-processed LMFP electrode formulations reported in Table 2. As visible in the figure, LMFP2 demonstrated the highest initial capacity (140 mAh·g^−1^) and higher overall capacity values at different C rates. As happened with LFP, working with Ketjenblack as a conductive additive and 1 wt.% of CMC in the binder solution resulted in the best cell performance. This effect results, similarly to LFP, from the cathode material structure, which normally presents both low electronic conductivity and low ionic diffusion [31,33,61].

Nevertheless, the cell performance in all cases was stable at low C rates, and it became unstable when the C rate increased. In addition, low initial coulombic efficiency (Figure 9b) values were obtained for all LMFP samples (LMFP1 73.6%, LMFP2 48.8%, LMFP3 56%, and LMFP4 74%). The LMFP3 sample showed a slightly better stability of coulombic efficiency, however, at the cost of capacity. The poor performance of the LMFP electrodes in the LiPCP electrolytes can be attributed to the fact that the electrochemical stability limit of the electrolyte prevents the full utilization of the electrode capacity, especially at higher rates. With the higher polarization of the cell than that of the LiPF_6_-based electrolyte, cells with LiPCP reach their cut-off potential at a lower capacity. It cannot be prevented by the increase in cut-off potential, due to the electrochemical stability limit of the electrolyte. It can be also attributed to the manganese characteristics and working in an aqueous system for such a compound; moreover, it possibly tends to dissolve easily, causing a rapid loss of capacity during the charge–discharge process.

**Figure 10:**
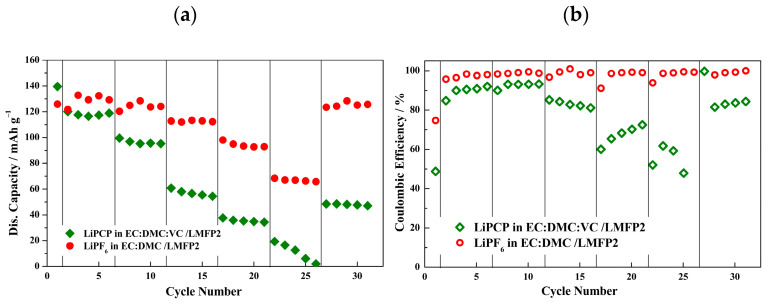
Rate performance of Li/LiMn_0.6_Fe_0.4_PO_4_ (LMFP2) cells with 0.8 mol·kg^−1^ LiPCP in EC:DMC (30:70 wt.%) and 5 wt.% of VC; and with 1.0 mol·kg^−1^ LiPF_6_ in EC:DMC. Potential range: 2.4–4.2 V vs. Li^+^/Li. (**a**) Discharge capacities in mAh·g^−1^. (**b**) Coulombic efficiency in percentage (%).

Furthermore, the performance of the best electrode (LMFP2) was compared between the LiPCP-based and standard LiPF_6_-based electrolytes (Figure 10a). The initial discharge capacity and initial coulombic efficiency values (Figure 10b) for LiPF_6_ and LiPCP were 125 mAh·g^−1^ and 74.8% and 139.6 mAh·g^−1^ and 48.8%, respectively. The LiPCP-based electrolyte showed a significantly worse performance than the LiPF_6_-based one for the LMFP aqueous-processed electrode. The reasons behind this can be understood by looking at the charge–discharge curves (Figures S4 and S5). A progressive polarization of the sample can be observed, which translates to a shift of Mn^3+^/Mn^2+^ plateau to potentials beyond the stability limit of LiPCP. Also, significant SEI formation can be seen during the initial charge, which explains the low initial coulombic efficiency. Nevertheless, the results give evidence for the need of further work on electrode and electrolyte optimization for this specific LMFP material. The LiPCP itself is not incompatible with the LMFP, however, in order to fully utilize its potential, the electrolyte composition causing lower polarization would be required. In addition, it is worth mentioning that metallic lithium was used as an anode, which may not be the most appropriate, as observed in the lithium passivation experiments.

### 2.5. Cycling Stability

**Figure 11:**
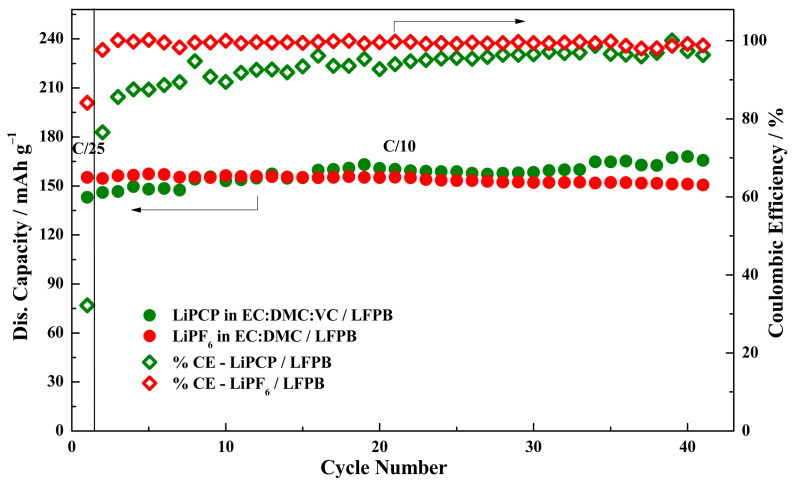
Cycling stability and coulombic efficiency at C/10 of Li/LiFePO_4_ (LFPB) cells with 0.8 mol·kg^−1^ LiPCP in EC:DMC (30:70 wt.%) and 5 wt.% of VC; and with 1.0 mol·kg^−1^ LiPF_6_ in EC:DMC. Potential range: 2.5–3.9 V vs. Li^+^/Li. The first formation cycle was performed at C/25.

**Figure 12:**
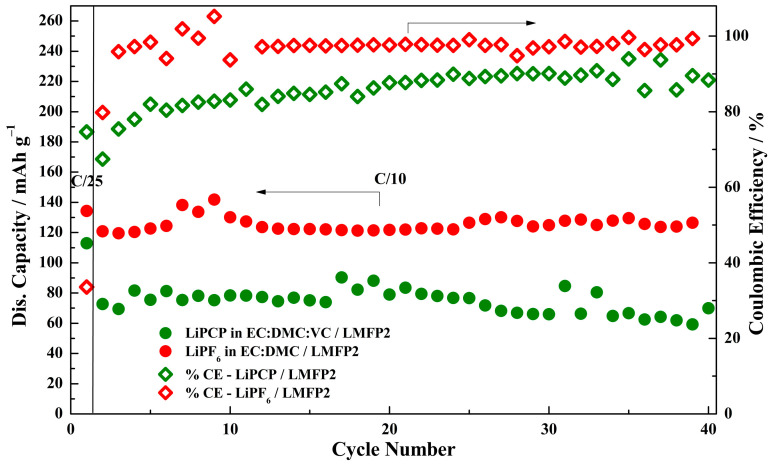
Cycling stability and coulombic efficiency at C/10 of Li/LiMn_0.6_Fe_0.4_PO_4_ (LMFP2) cells with 0.8 mol·kg^−1^ LiPCP in EC:DMC (30:70 wt.%) and 5 wt.% of VC; and with 1.0 mol·kg^−1^ LiPF_6_ in EC:DMC. Potential range: 2.4–4.2 V vs. Li^+^/Li. The first formation cycle was performed at C/25.

The cycling stability was determined for the optimized LFPB (Figure 11) and LMFP2 (Figure 12) electrodes with the LiPCP-based electrolyte and the standard LiPF_6_-based one during C/10 cycling. Starting with LFP, the performance of both samples was similar, with LiPCP having a slightly lower initial capacity (143 vs. 155 mAh·g^−1^), which increased over time to reach a stable value for LiPF_6_ after 15 cycles. This increase in capacity cannot be attributed to parasitic reactions, as the coulombic efficiency of the LiPCP sample also increased with time to the value of ca. 99%. However, the lower capacity in the first 10 cycles can be attributed to the wettability, since this cell had only 4 h of resting time before cycling. When it comes to LMFP, the results are worse for LiPCP. Although the capacity does not decrease significantly during the measurement, it oscillates around a mere 65–70 mAh·g^−1^ at a rate of only C/10. It is significantly lower than the stable ca. 120 mAh·g^−1^ obtained for the LiPF_6_-based electrolyte. The relative stability of capacity values for both samples indicates that the problem does not lie in the electrode, and that further electrolyte adjustment should be carried out, especially in the case of LMFP. Such adjustments should provide a reduction in the overpotentials in the cell. With lowered overpotentials, it might be feasible to combine the LiPCP-based electrolyte with the LMFP cathode, utilizing its full capacity within the existing potential limitations.

## 3. Materials and Methods

### 3.1. Reagents

Ethylene carbonate (EC), dimethyl carbonate (DMC), ethyl-methyl carbonate (EMC), diethyl carbonate (DEC), and vinylene carbonate (VC) battery-grade solvents were purchased from BASF (Ludwigshafen am Rhein, Germany). Lithium hexafluorophosphate (LiPF_6_, battery grade) and acetonitrile (AN, anhydrous, 99.98%) were obtained from Sigma Aldrich (Saint Luis, MO, USA). A total of 1 mol·kg^−1^ LiPF_6_ in EC:DMC (30:70 wt.%) was purchased from E-Lyte Innovations (battery grade). The metallic lithium foil was purchased from Honjo Metal. The lithium iron phosphate (LFP, LiFePO_4_, 1.45 wt.% carbon) and lithium iron manganese phosphate (LMFP, LiMn_0.6_Fe_0.4_PO_4_, 1.5~2.5 wt.% carbon) powdered materials were acquired from MTI (Richmond, CA, USA) and MSE Supplies, respectively. Sodium carboxymethyl cellulose (CMC, average M_w_ of 250,000, degree of substitution equal to 0.9) was purchased from Sigma Aldrich. Super-P and Ketjenblack conductive carbons were procured from Alfa Aesar and MSE Supplies, respectively. The carbon-coated aluminum foil (15 µm thickness of aluminum, 1 μm of conductive carbon) was purchased from MSE Supplies.

### 3.2. Electrolyte Preparation

The LiPCP salt was synthesized in the laboratory at the Warsaw University of Technology in a two-step procedure, as reported elsewhere [23]. First, a stoichiometric amount of water and excess pyridine was added to tetracyanoethylene to obtain pyridinium pentacyanopropenide. Afterwards, tetrahydrofuran (THF) and lithium hydride (LiH) were added as the monovalent metal donor. After filtration and washing with ether at room temperature, LiPCP was obtained. LiPCP salt has a purity above 99%, and the characterization by nuclear magnetic resonance (NMR) is shown as follows:

^13^C NMR (125 MHz; acetone–d_6_) δ/ppm: 135,7 (C1–C2–C3), 117,0 (2C, C1–CN/C3–CN (the side group of the three cyano groups that are on one side of the molecule)), 114,6 (C2–CN), 113,9 (2C, C1–CN/C3–CN (two cyano groups on one side of the molecule)), 57,8 (2C, C1–C2–C3), [23].

The fluorine-free electrolyte solutions were prepared in an argon-filled glovebox (LabStar, MBraun, H_2_O < 1 and O_2_ < 10 ppm, Garching, Germany)) by dissolving the LiPCP salt in different solvents. For solvent screening, electrolyte solutions of 0.8 mol·kg^−1^ LiPCP in EC:DMC (30:70 wt.%), EC:EMC (30:70 wt.%), and EC:DEC (30:70 wt.%) were prepared. For comparison, the commercial standard 1 mol·kg^−1^ LiPF_6_ electrolyte solutions were prepared in the same solvent mixtures. Additionally, for molality screening, solutions from 0.1 to 1.2 mol·kg^−1^ LiPCP in EC:DMC (30:70 wt.%) were prepared.

### 3.3. Ionic Conductivity Measurements

The ionic conductivity of the LiPCP-based electrolytes was obtained via electrochemical impedance spectroscopy (EIS) using a VMP3 potentiostat-galvanostat (VMP3, Bio-Logic, Seyssinet-Pariset, France) in the temperature range of 0 °C to 50°C. The measurements were carried out in the frequency range of 500 kHz to 1 Hz with 10 points per decade and a signal amplitude of 5 mV. The measurement at each frequency was repeated 6 times. The conductivity micro cells consisted of an electrolyte placed between two stainless steel electrodes. A cryostat-thermostat (Haake K75), (Vreden, Germany) with a temperature controller (DC50) was used for the thermoset samples.

The conductivity (σ) in mS·cm^−1^ was calculated according to Equation (1), as follows:

(1) $$\sigma =\frac{k}{R}$$

where k is the cell constant (0.3–0.7 cm^−1^, determined with ± 0.3% precision) for each conductivity microcell and R is the bulk resistance in ohms (Ω) obtained from the respective Nyquist plot.

### 3.4. Linear Sweep Voltammetry

Linear sweep voltammetry (LSV) was used to evaluate the electrochemical stability window of the following electrolytes: 0.8 mol·kg^−1^ LiPCP in EC:DMC (30:70 wt.%), 0.8 mol·kg^−1^ LiPCP in EC:DMC (30:70 wt.%) + 5 wt.% VC, 0.8 mol·kg^−1^ LiPCP in EC:DMC (30:70 wt.%) + 5 wt.% VC + 5 wt.% AN, and 0.8 mol·kg^−1^ LiPCP in EC:DMC (30:70 wt.%) + 5 wt.% VC + 10 wt.% AN. The tests were conducted at room temperature in a two-electrode Swagelok-type cell configuration at a scan rate of 0.5 mV s^−1^. For the set-up, a Pt disc was used as the working electrode and a Li metal disc as the counter and reference electrode.

### 3.5. Lithium Passivation Measurement

Lithium passivation was investigated with electrochemical impedance spectroscopy (EIS) for the following electrolytes: 0.8 mol·kg^−1^ LiPCP in EC:DMC (30:70 wt.%) + 5 wt.% VC, 0.8 mol·kg^−1^ LiPCP in EC:DMC (30:70 wt.%) + 5 wt.% VC + 10 wt.% AN, and 1.0 mol·kg^−1^ LiPF_6_ in EC:DMC (30:70 wt.%). The measurements were carried out in the Swagelok-type cells with two lithium electrodes (discs) with a Celgard 2400 separator in between of them. For the measurement, a VMP3 potentiostat-galvanostat (VMP3, Bio-Logic) was used. The measurements were carried out in the frequency range of 500 kHz to 1 Hz, with 10 points per decade and signal amplitude of 5 mV. The measurement at each frequency was repeated 6 times. The impedance spectra were repeatedly measured in a period of 6 h. The Impedance spectra were analyzed using RelaxIS 3 software.

### 3.6. Electrode Preparation

LFP and LMFP were used independently as the active materials (AM). CMC was employed as the binder. Super-P and Ketjenblack conductive carbon black were used independently as the conductive additives (CMs) for electrode preparation. The components were mixed following different AM:CM:binder ratios (as indicated where appropriate throughout the manuscript) by using magnetic stirring (IKA-type RH Basic Magnetic Stirrer, Staufen im Breisgau, Germany) at 500 rpm for 24 h, and distilled water as the solvent. The obtained slurry was then cast onto carbon-coated aluminum foil by using a blade coater (Doctor Blade, ZEHNTNER Testing Instruments, ZAA 2300, and ZEHNTNER film applicator ZUA 2000, Sissach, Switzerland). All electrodes were first dried at 80 °C for 2 h, then, subsequently, they were vacuum dried at 120 °C overnight (Memmert VO 400, Schwabach, Germany)).

### 3.7. Cyclic Voltammetry Measurement

To evaluate the electrode/electrolyte compatibility, cyclic voltammetry (CV) measurements were conducted in a potentiostat-galvanostat (VMP3, Bio-Logic). LFP and LMFP electrodes of 11 mm in diameter were punched and assembled in two-electrode Swagelok-type cell configuration using a disc of Li metal as a counter electrode. Celgard 2400 microporous polypropylene was used as the separator, 0.8 mol·kg^−1^ LiPCP in EC:DMC (30:70 wt.%) was used as the electrolyte, and 1 mol·kg^−1^ LiPF_6_ in EC:DMC (30:70 wt.%) was used as the reference electrolyte. The scan rate was set at 0.5 mV·s^−1^ in 2.5–4.0 V vs. Li^+^/Li potential limits for the LFP cathode and 2.5–4.5 V vs. Li^+^/Li potential limits for the LMFP cathode.

### 3.8. Galvanostatic Cycling

For galvanostatic cycling, LFP and LMFP electrodes of 15 mm in diameter were punched and assembled in CR2032 coin cells, using a Li metal disc as the negative electrode and Celgard 2400 as the separator. The electrolyte solution was 0.8 mol·kg^−1^ LiPCP in EC:DMC (30:70 wt.%) + 5 wt.% VC. The tests were performed in a SOLLICH potentiostat-galvanostat (SOLLICH 2061 MPG&T, Multichannel Potentiostat-Galvanostat and Battery Tester) in 2.5–3.9 V vs. Li^+^/Li potential limits for the LFP cathode and 2.4–4.2 V vs. Li^+^/Li for the LMFP cathode. The protocol consisted of one formation cycle at C/25 (where C = 170 mAh·g^−1^ in the case of LFP and 160 mAh·g^−1^ in the case of LMFP) followed by C/10 cycling. In addition, the rate capability of the LFP and LMFP cathodes with the LiPCP-based electrolyte was screened. To this purpose, 1 cycle at C/25, 5 cycles at C/20, 5 cycles at C/10, 5 cycles at C/5, 5 cycles at C/2, and 5 cycles at 1C were performed.

Table 4 shows the various electrolyte compositions used in the different experiments throughout this research.

## 4. Conclusions

In summary, the electrochemical performance of aqueous-processed olivine-type LFP and LMFP electrodes was systematically investigated using a fluorine-free LiPCP-based electrolyte. The precise optimization of the electrolyte composition revealed that an EC:DMC (30:70 wt.%) mixture provided the highest ionic conductivity. The optimal concentration of LiPCP was found to be 0.8 mol·kg^−1^, achieving a conductivity of 9.6 mS·cm^−1^ at 20 °C. This electrolyte exhibited electrochemical stability up to 4.4 V vs. Li^+^/Li when VC was utilized as a solid electrolyte interphase (SEI)-stabilizing additive, resulting in a fully fluorine-free electrolyte with favorable characteristics.

A comprehensive screening of various compositions for aqueous-processed LFP and LMFP electrodes was conducted. The optimal component ratio was found to be 87:10:3 (AM:CM:binder). Two conductive additives, Super P and Ketjenblack, were evaluated, with Ketjenblack consistently delivering a superior electrochemical performance. The concentration of CMC in the binder solution during electrode preparation was varied, and a concentration of 1% yielded the most favorable electrode properties. The selected electrode formulation proved suitable, achieving specific capacities of 150 mAh·g^−1^ (LFP) and 125 mAh·g^−1^ (LMFP) with the reference LiPF_6_ electrolyte.

LiPCP-based electrolytes, incorporating fluorine-free linear carbonates as solvents and a stabilizing additive, exhibited fast kinetics and stable reversible cycling. Stable galvanostatic cycling of half-cells confirmed the compatibility between the LiPCP and olivine-type cathodes. The electrochemical performance of LFP with LiPCP was comparable to that with LiPF_6_. On the contrary, the performance of LMFP with LiPCP was inferior, attributed to increased cell polarization, which hindered the full utilization of the high-potential plateau of this cathode. While LiPCP is compatible with LMFP, further optimization of the electrolyte composition for this system is necessary.

In conclusion, lithium-ion batteries devoid of critical and problematic elements such as cobalt, nickel, and fluorine were engineered and exhibited a satisfactory performance. These findings represent the initial phase of a groundbreaking investigation into developing safer and more environmentally friendly electrolytes compatible with aqueous-processed electrodes for lithium-ion batteries. This first stage lays the foundation for a novel approach, aiming to innovate the battery industry by reducing its dependence on hazardous materials and assisting in improving the overall sustainability and safety of energy storage systems.

## Figures and Tables

**Table 1:** Weight compositions (LiFePO_4_, Super P, and CMC binder) and key results of various LFP-based cathodes used in cyclic voltammetry experiments.

Code	Active Material (AM)/wt.%, LFP	Conductive Material (CM)/wt.%, Super P	Binder/ wt.%, CMC	Oxidation Peak/V	Reduction Peak/V	ΔV/V	Discharge Peak Current Density/mA·cm^−2^
LFP1	85	10	5	3.94	3.21	0.73	0.33
LFP2	87	10	3	3.88	3.23	0.65	1.08
LFP3	88	10	2	3.84	3.23	0.61	0.13
LFP4	87.5	10	2.5	3.94	3.14	0.80	0.85
LFP5	90	8	2	3.91	3.24	0.68	0.80
LFP6	90	7.5	2.5	3.90	3.24	0.65	0.61
LFP7	90	7	3	3.91	3.26	0.65	0.47
LFP8	80	17	3	3.91	3.21	0.71	0.83
LFP9	83	14	3	3.92	3.17	0.75	0.25
LFP10	85	12	3	3.92	3.25	0.67	0.64

**Table 2:** Weight compositions (LiMn_0.6_Fe_0.4_PO_4_, conductive material, and CMC binder) and key results of various LMFP-based cathodes used in cyclic voltammetry.

Code	Active Material (AM)/ wt.%. LMFP	Conductive Material (CM)/wt.%	Binder /wt.% CMC	Conductive Material Carbon Black Type	Concentration CMC in Solution (wt.%)	Oxidation Peak/V	Reduction Peak/V	ΔV/V	Discharge Peak Current Density/mA·cm^−2^
LMFP1	87	10	3	Super P	1.0	3.83	3.36	0.47	0.38
LMFP2	87	10	3	Ketjenblack EC600JD	1.0	3.81	3.26	0.55	0.77
LMFP3	87	10	3	Super P	1.5	3.86	3.33	0.53	0.54
LMFP4	87	10	3	Ketjenblack EC600JD	1.5	3.81	3.32	0.49	0.67

**Table 3:** LFP cathode slurry composition for galvanostatic charge/discharge cycling.

Code	Active Material (AM)/wt.% LFP	Conductive Material (CM)/wt.%	Binder /wt.% CMC	Conductive Material Carbon Black—Type	Concentration of CMC in Solution/wt.%
LFPA	87	10	3	Super P	1.0
LFPB	87	10	3	Ketjenblack EC600JD	1.0
LFPC	87	10	3	Super P	1.5
LFPD	87	10	3	Ketjenblack EC600JD	1.5

**Table 4:** Electrolyte compositions used in the different experiments.

Experiment	Electrolyte	Observations
Ionic conductivity measurements	0.1 to 1.2 mol·kg^−1^ LiPCP in EC:DMC (30:70 wt.%)	Screening of compositions at the temperature range of 0 °C to 50 °C
	0.8 mol·kg^−1^ LiPCP in EC:DMC (30:70 wt.%) EC:EMC (30:70 wt.%) EC:DEC (30:70 wt.%)	Comparison of conductivities of LiPCP and the most commercial electrolyte LiPF_6_ in different organic carbonate solvents
	1 mol·kg^−1^ LiPF_6_ in EC:DMC (30:70 wt.%) EC:EMC (30:70 wt.%) EC:DEC (30:70 wt.%)	
Linear sweep voltammetry	0.8 mol·kg^−1^ LiPCP in EC:DMC (30:70 wt.%) 0.8 mol·kg^−1^ LiPCP in EC:DMC (30:70 wt.%) + 5 wt.% VC 0.8 mol·kg^−1^ LiPCP in EC:DMC (30:70 wt.%) + 5 wt.% VC + 5 wt.% AN 0.8 mol·kg^−1^ LiPCP in EC:DMC (30:70 wt.%) + 5 wt.% VC + 10 wt.% AN	Evaluating the electrochemical stability window
Lithium passivation	0.8 mol·kg^−1^ LiPCP in EC:DMC (30:70 wt.%) + 5 wt.% VC 0.8 mol·kg^−1^ LiPCP in EC:DMC (30:70 wt.%) + 5 wt.% VC + 10 wt.% AN 1.0 mol·kg^−1^ LiPF_6_ in EC:DMC (30:70 wt.%)	Evaluating the lithium effect and effect in the cycling
Galvanostatic charge/discharge cycling	0.8 mol·kg^−1^ LiPCP in EC:DMC (30:70 wt.%)	This composition was used for the cycling, compatibility, and stability evaluation

## Data Availability

The data presented in this study are available in article and Supplementary Materials.

## Supplementary Materials

The following supporting information can be downloaded at: https://www.mdpi.com/article/10.3390/molecules29194698/s1, Table S1. Conductivity of LiPCP and LiPF_6_ at different temperatures and carbonate mixture solvents; Table S2. Percentage difference in LiPCP and LiPF_6_ conductivities; Table S3. Conductivity of LiPCP at different concentrations; Figure S1. (**a**) Nyquist plots of 0.8 mol·kg^−1^ LiPCP in EC:DMC (30:70 wt.%) with 5 wt.% of VC, (**b**) Nyquist plots of 0.8 mol·kg^−1^ LiPCP in EC:DMC (30:70 wt.%) with 5 wt.% of VC + 10 wt.% of AN, (**c**) Nyquist plots of LiPF_6_ in ED:DMC (30:70 wt.%), (**d**) Equivalent circuit used for analysis of impedance spectra; Figure S2. Charge–discharge curves at different C rates for LiPCP in EC:DMC (30:70 wt.%) + 5 wt.%VC, cell Li/LFPB; Figure S3. Charge–discharge curves at different C rates for LiPF_6_ in EC:DMC (30:70 wt.%), cell Li/LFPB; Figure S4. Charge–discharge curves at different C rates for LiPCP in EC:DMC (30:70 wt.%) + 5 wt.%VC, cell Li/LMFP2; Figure S5. Charge–discharge curves at different C rates for LiPF_6_ in EC:DMC (30:70 wt.%), cell Li/LMFP2.
