# Supplementary material for: Development of Fluorine-Free Electrolytes for Aqueous-Processed Olivine-Type Phosphate Cathodes
Source: Molecules. 2024 Oct 4;29(19):4698. doi: 10.3390/molecules29194698 (PMC11477786; doi:10.3390/molecules29194698)
Supplement: Supplementary file 1 [file molecules-29-04698-s001.zip › molecules-3227128-supplementary.pdf]

# SUPPORTING INFORMATION

## DEVELOPMENT OF FLUORINE-FREE ELECTROLYTES FOR AQUEOUS-PROCESSED OLIVINE-TYPE PHOSPHATE CATHODES

Claudia Limachi[a,b,c,*], Klaudia Rogala[a], Marek Broszkiewicz[a], Marta Cabello[b], Leszek Niedzicki[a,c,*], Michel Armand[b], Władysław Wieczorek[a,c]

[a] Faculty of Chemistry, Warsaw University of Technology, 00-664 Warsaw, Poland
[b] Centre for Cooperative Research on Alternative Energies (CIC energiGUNE), Basque Research and Technology Alliance (BRTA), Alava Technology Park, Albert Einstein 48, 01510 Vitoria-Gasteiz, Spain
[c] Alistore-European Research Institute, CNRS FR 3104, Hub de l'Énergie, Amiens, 80039, France
[*] Corresponding author

**Table S1. Conductivity of LiPCP and $LiPF_6$ at different temperatures and carbonate mixture solvents**

| Electrolyte ($mol\cdot Kg^{-1}$) | $\kappa/mS\cdot cm^{-1}$ (0°C) | $\kappa/mS\cdot cm^{-1}$ (10°C) | $\kappa/mS\cdot cm^{-1}$ (20°C) | $\kappa/mS\cdot cm^{-1}$ (30°C) | $\kappa/mS\cdot cm^{-1}$ (40°C) | $\kappa/mS\cdot cm^{-1}$ (50°C) |
|---|---|---|---|---|---|---|
| 0.8 LiPCP in 3EC:7DMC | 6.48 | 7.99 | 9.63 | 11.38 | 13.41 | 15.02 |
| 0.8 LiPCP in 3EC:7DEC | 2.92 | 3.69 | 4.48 | 5.46 | 6.44 | 7.38 |
| 0.8 LiPCP in 3EC:7EMC | 4.39 | 5.35 | 6.36 | 7.57 | 8.78 | 10.21 |
| 1.0 $LiPF_6$ in 3EC:7DMC | 8.16 | 10.13 | 12.30 | 14.52 | 16.91 | 19.58 |
| 1.0 $LiPF_6$ in 3EC:7DEC | 3.89 | 4.95 | 6.14 | 7.41 | 8.87 | 10.48 |
| 1.0 $LiPF_6$ in 3EC:7EMC | 5.45 | 6.74 | 7.76 | 8.83 | 9.85 | 11.13 |

**Table S2. % Difference of LiPCP and $LiPF_6$ conductivities**

| %Difference of $LiPF_6$/LiPCP | T / °C | | | | | |
|---|---|---|---|---|---|---|
| | 0 °C | 10 °C | 20 °C | 30 °C | 40 °C | 50 °C |
| % Diff. EC:DMC | 25.95 | 26.82 | 27.75 | 27.60 | 26.03 | 30.35 |
| % Diff. EC:DEC | 33.45 | 34.21 | 37.05 | 35.87 | 37.76 | 41.93 |
| % Diff. EC:EMC | 24.07 | 25.84 | 21.93 | 16.63 | 12.14 | 9.02 |

**Table S3. Conductivity of LiPCP at different concentrations**

| Concentration (mol·Kg$^{-1}$) LiPCP [3EC/7DMC] | κ/mS·cm$^{-1}$ (0°C) | κ/mS·cm$^{-1}$ (10°C) | κ/mS·cm$^{-1}$ (20°C) | κ/mS·cm$^{-1}$ (30°C) | κ/mS·cm$^{-1}$ (40°C) | κ/mS·cm$^{-1}$ (50°C) |
|---|---|---|---|---|---|---|
| 0.1 | 2.29 | 2.75 | 3.20 | 3.65 | 4.03 | 4.47 |
| 0.2 | 3.28 | 3.96 | 4.54 | 5.01 | 5.75 | 6.54 |
| 0.3 | 3.43 | 4.09 | 4.77 | 5.54 | 6.31 | 7.15 |
| 0.4 | 4.77 | 5.79 | 6.80 | 7.82 | 8.75 | 9.76 |
| 0.5 | 4.83 | 5.63 | 6.31 | 7.23 | 8.37 | 9.75 |
| 0.6 | 6.19 | 7.35 | 8.84 | 10.06 | 11.56 | 12.76 |
| 0.7 | 5.84 | 7.36 | 8.58 | 9.88 | 11.73 | 13.54 |
| 0.8 | 6.48 | 7.99 | 9.63 | 11.38 | 13.41 | 15.02 |
| 0.9 | 4.80 | 6.05 | 7.47 | 8.81 | 10.31 | 12.04 |
| 1.0 | 4.93 | 6.37 | 7.97 | 9.61 | 11.41 | 13.29 |
| 1.1 | 3.02 | 4.42 | 5.85 | 7.28 | 8.67 | 10.51 |
| 1.2 | 4.43 | 5.82 | 7.39 | 8.82 | 10.69 | 12.35 |

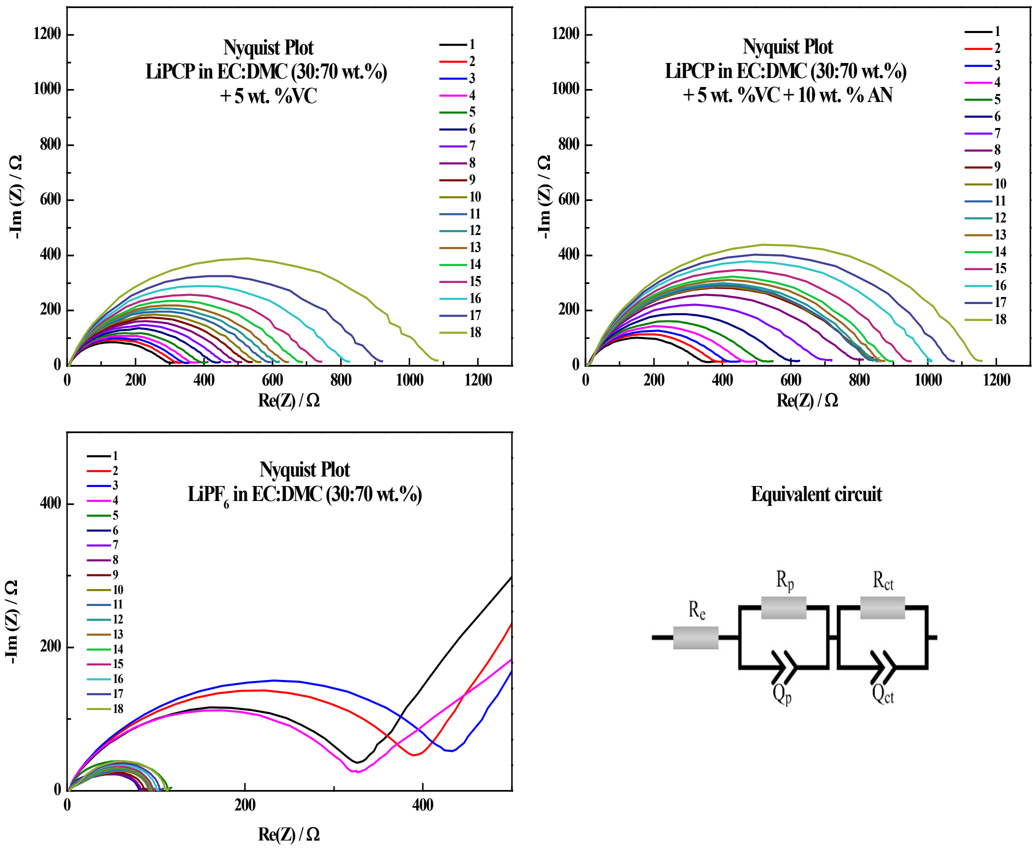


**Figure S1. a) Nyquist Plots of 0.8 mol·kg$^{-1}$ LiPCP in EC:DMC (30:70 wt.%) with 5 wt.% of VC, b) Nyquist Plots of 0.8 mol·kg$^{-1}$ LiPCP in EC:DMC (30:70 wt.%) with 5 wt.% of VC + 10 wt.% of AN, c) Nyquist Plots of $LiPF_6$ in ED:DMC (30:70 wt.%), d) Equivalent circuit used for analysis of impedance spectra.**

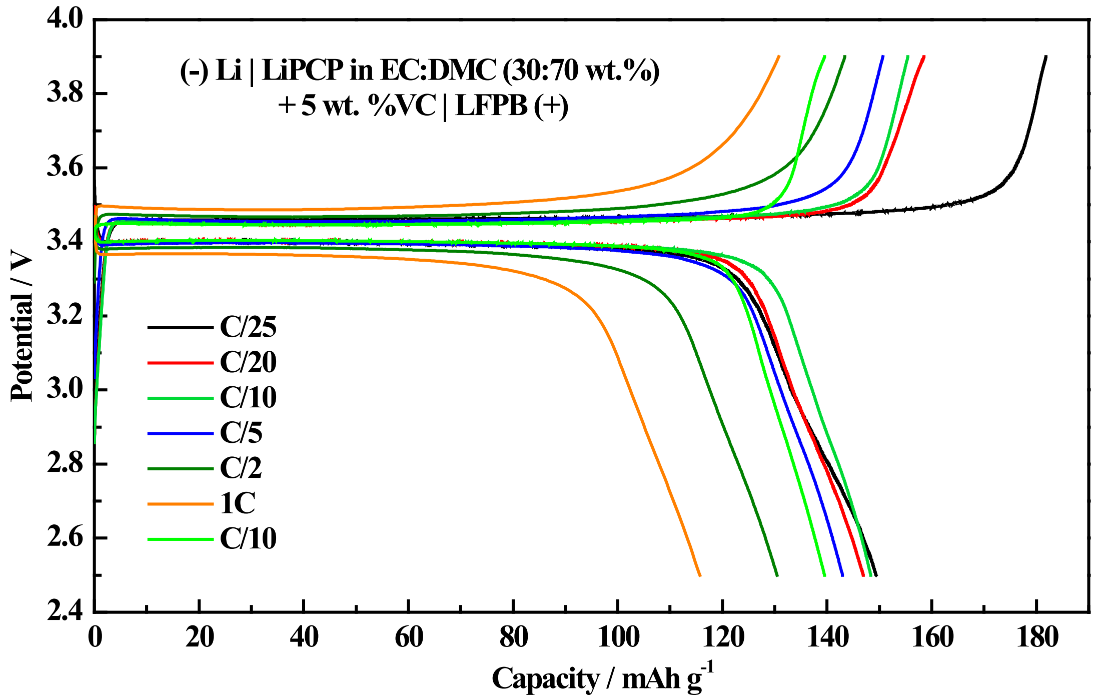


**Figure S2. Charge-discharge curves at different C-rates for LiPCP in EC:DMC (30:70 wt.%) + 5 wt. %VC, cell Li/LFPB.**

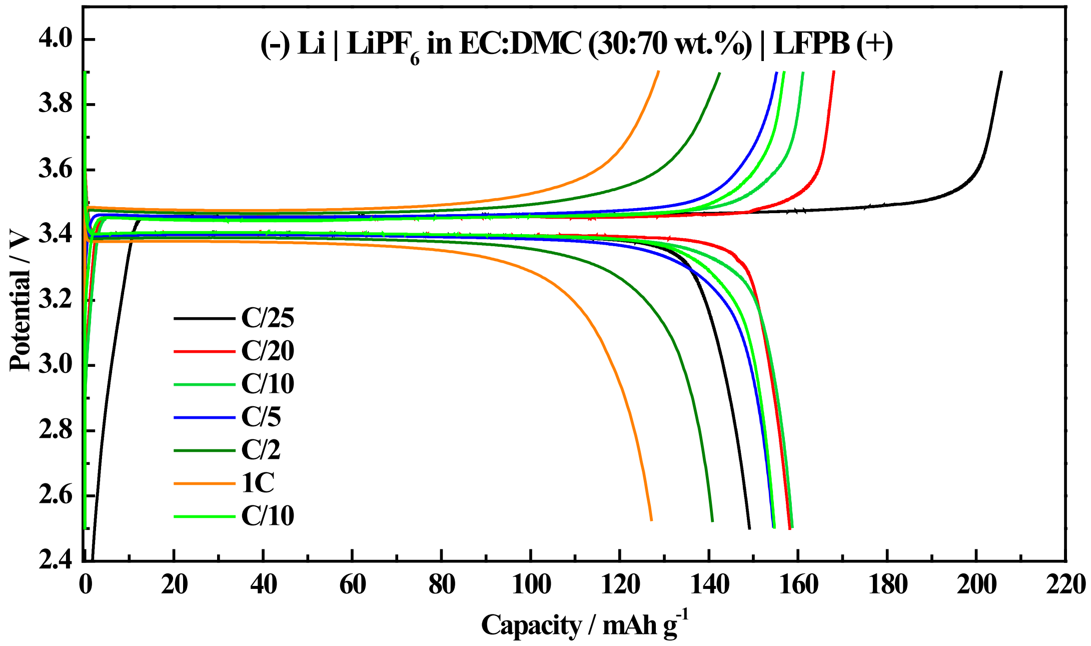


**Figure S3. Charge-discharge curves at different C-rates for $LiPF_6$ in EC:DMC (30:70 wt.%), cell Li/LFPB.**

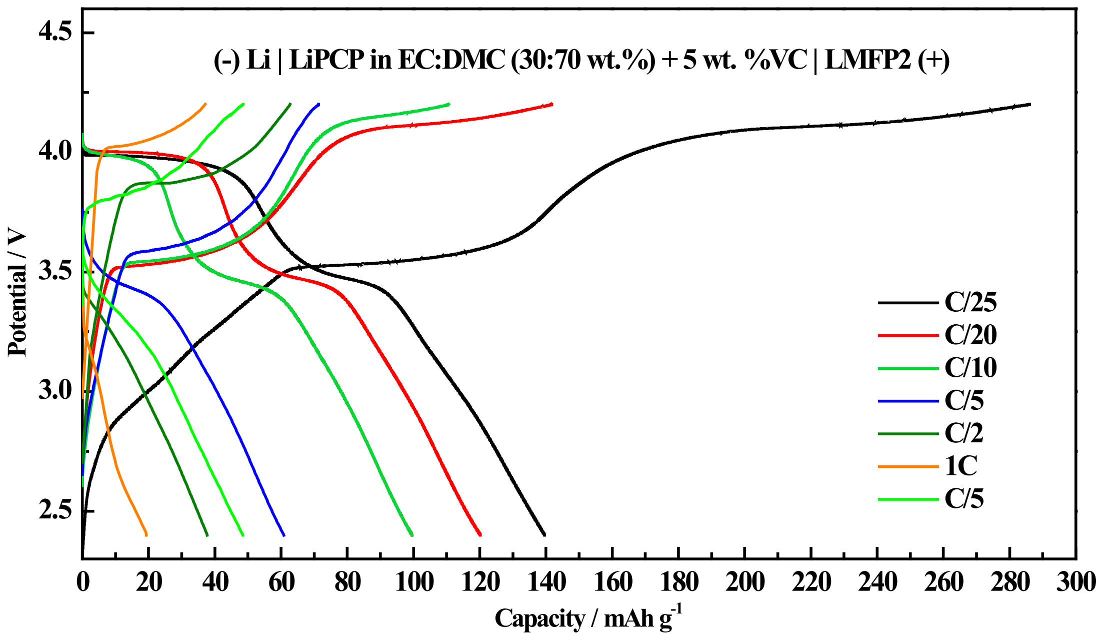


**Figure S4. Charge-discharge curves at different C-rates for LiPCP in EC:DMC (30:70 wt.%) + 5 wt. %VC, cell Li/LMFP2.**

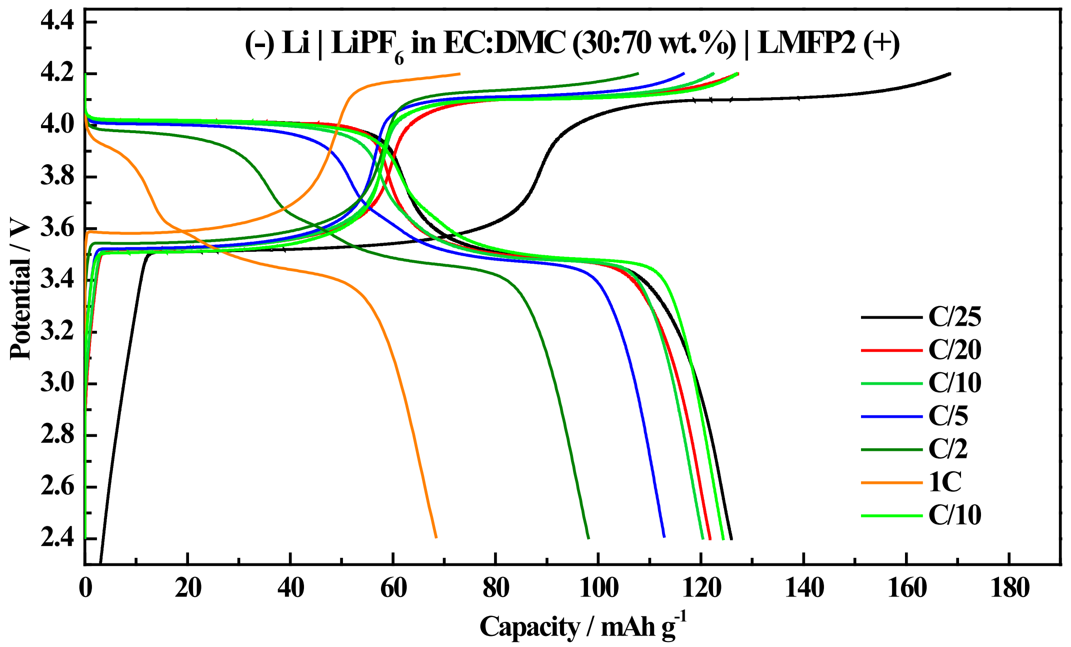


**Figure S5. Charge-discharge curves at different C-rates for $LiPF_6$ in EC:DMC (30:70 wt.%), cell Li/LMFP2.**
